# Factors associated with macrosomia, hypoglycaemia and low Apgar score among Fijian women with gestational diabetes mellitus

**DOI:** 10.1186/s12884-020-2821-6

**Published:** 2020-02-28

**Authors:** Falahola Fuka, Uchechukwu L. Osuagwu, Kingsley Agho, Rajat Gyaneshwar, Swaran Naidu, James Fong, David Simmons

**Affiliations:** 1 Ministry of Health, Vaoila Hospital, Nuku’alofa, Tongatapu Tonga; 2 Diabetes, Obesity and Metabolism Translational Research Unit (DOMTRU), School of Medicine, Western Sydney University, Campbelltown, 2560 NSW Australia; 3 School of Health Sciences, Western Sydney University, Campbelltown, 2560 NSW Australia; 4 African Vision Research Institute, University of KwaZulu-Natal Durban, Durban, South Africa; 5 Department of Obstetrics and Gynaecology, Lautoka Hospital, Lautoka, Fiji; 6 Department of Obstetrics and Gynecology, CWM Hospital, Suva, Fiji

**Keywords:** Gestational diabetes mellitus (GDM), Pregnancy, Fiji, Pacific people, Hypoglycaemia, Macrosomia, Apgar score, Diabetes

## Abstract

**Background:**

Gestational diabetes mellitus (GDM) in Fiji is a serious public health issue. However, there are no recent studies on GDM among pregnant women in Fiji. The aim of this study was to examine prevalence of, and sociodemographic factors associated with adverse neonatal outcomes among Fijian women with GDM.

**Methods:**

We used cross-sectional data of 255 pregnant women with GDM who gave birth to singleton infants at Colonial War Memorial Hospital (CWMH) in Suva city. Women underwent testing for GDM during antenatal clinic visits and were diagnosed using *modified* International Association of Diabetes and Pregnancy Study Groups (IADPSG) criteria. Multivariable logistic regression analysis was used to investigate factors associated with neonatal outcomes.

**Results:**

Women with a previous baby weighing > 4 kg were 6.08 times more likely to experience neonatal macrosomia (Adjusted odds ratio (AOR) = 6.08; 95%CI: 2.46, 15.01). Compared to unmarried women, the odds of macrosomia among married women reduced by 71% (AOR = 0.29; 95%CI: 0.11, 0.77). Compared with delivery before 38 weeks of gestation, the infants of women who delivered between 38 and 41 weeks of gestation were 62 and 86% less likely to experience neonatal hypoglycaemia and Apgar score < 7 at 5 mins, respectively. The offspring of women who were overweight and obese had higher odds of neonatal hypoglycaemia. Late booking in gestation (≥28 weeks) was significantly associated with Apgar score < 7 at 5 min (AOR = 7.87; 95%CI: 1.11, 55.75). Maternal pre-eclampsia/pregnancy induced hypertension was another factor associated with low Apgar score in infants.

**Conclusions:**

The study found high rates of adverse neonatal outcomes among off springs of Fijian women with GDM and showed that interventions targeting pregnant women who are overweight, had a previous baby weighing > 4 kg, had pre-eclampsia, delivered before 38 weeks of gestation, and those who booked later than 13 weeks in gestation, are needed to improve pregnancy outcomes.

## Introduction

Gestational diabetes mellitus (GDM) is any degree of glucose intolerance that occurs or is diagnosed for the first time during pregnancy [1]. Women with GDM are at high risk of pregnancy complications, including infant macrosomia, neonatal hypoglycaemia, low Apgar score and caesarean delivery [2], have more than a 7-fold increased risk of developing type 2 diabetes 5 to 10 years after delivery [3], and the risk is even higher in obese women with GDM [4]. Children born to mothers with GDM are more likely to develop impaired glucose tolerance later in life, [5] and early detection and appropriate therapy may prevent these complications [6, 7].

Globally, it is estimated that GDM affects between 1 and 36% of pregnancies, depending on the population studied and the diagnostic tests used [2]. Among Pacific people, previous studies conducted in 2008 reported that about 20% of pregnancies are complicated by GDM [8] but lower rates have been documented among European women living in Auckland who delivered between 1994 and 1995 [9], and White-skinned women in Hawaii, who delivered between 2010 and 2011 [10], compared with Pacific Island women. Lower rates of GDM were reported among Pacific Islands women living in Australia (6.3%) [11] and in the US (8.3%) [12], between 2010 and 2011 using the Australasian Diabetes in Pregnancy Society (ADIPS) [1] and the American Diabetes Association (ADA) diagnostic criteria [12]. Independent of maternal body weight, age, parity and education, women particularly those from Pacific countries who were born in their home countries were more likely to have GDM than their counterparts who were born in foreign countries [12]. The authors suggested that the varying degrees of access to medical care, especially recent immigrants who may be less likely to undergo screening for GDM, coupled with other environmental factors in migrant populations may interact with genetic susceptibility to influence the risk of GDM [12].

Fiji has high and increasing rates of obesity and type 2 diabetes among non-pregnant individuals [13] suggesting a high prevalence of gestational diabetes mellitus (GDM), and associated adverse pregnancy outcomes. Maternal obesity and type 2 diabetes were additive for increased risk of GDM [14]. However, there is no up-to-date evidence on the prevalence of, and outcomes from, pregnancies complicated by GDM in Fiji, and a meta-analysis failed to find reliable data on GDM in Pacific Island countries [15]. Prior epidemiological studies from Fiji were either conducted more than three decades ago (1983 [16] and 1990) [17], and/or used diagnostic criteria for GDM (O’Sullivan and Mahan 1964 [17] and WHO 1980 [16]) that are no longer in use. Gyaneshwar and Ram [17] found a higher prevalence of GDM among Fijians of Indian Descent (FIDs) compared with ITaukei Fijians (5% vs 0.6%) and the rate was higher among women who had higher BMI [17]. Zimmet et al. [16] found rural-urban differences in prevalence of diabetes in a Melanesian population with greater differences among women, but no difference was found between rural-urban dwellers among the Indian population. Since these studies (1989–2018), the rates of obesity and diabetes among Pacific Island countries [18] including Fiji [13] have more than doubled, and the criteria for GDM diagnosis in the previous studies were not designed to identify women at risk of adverse perinatal outcomes but to identify those at high risk of subsequently developing type 2 diabetes [19].

**Fig. 1:**
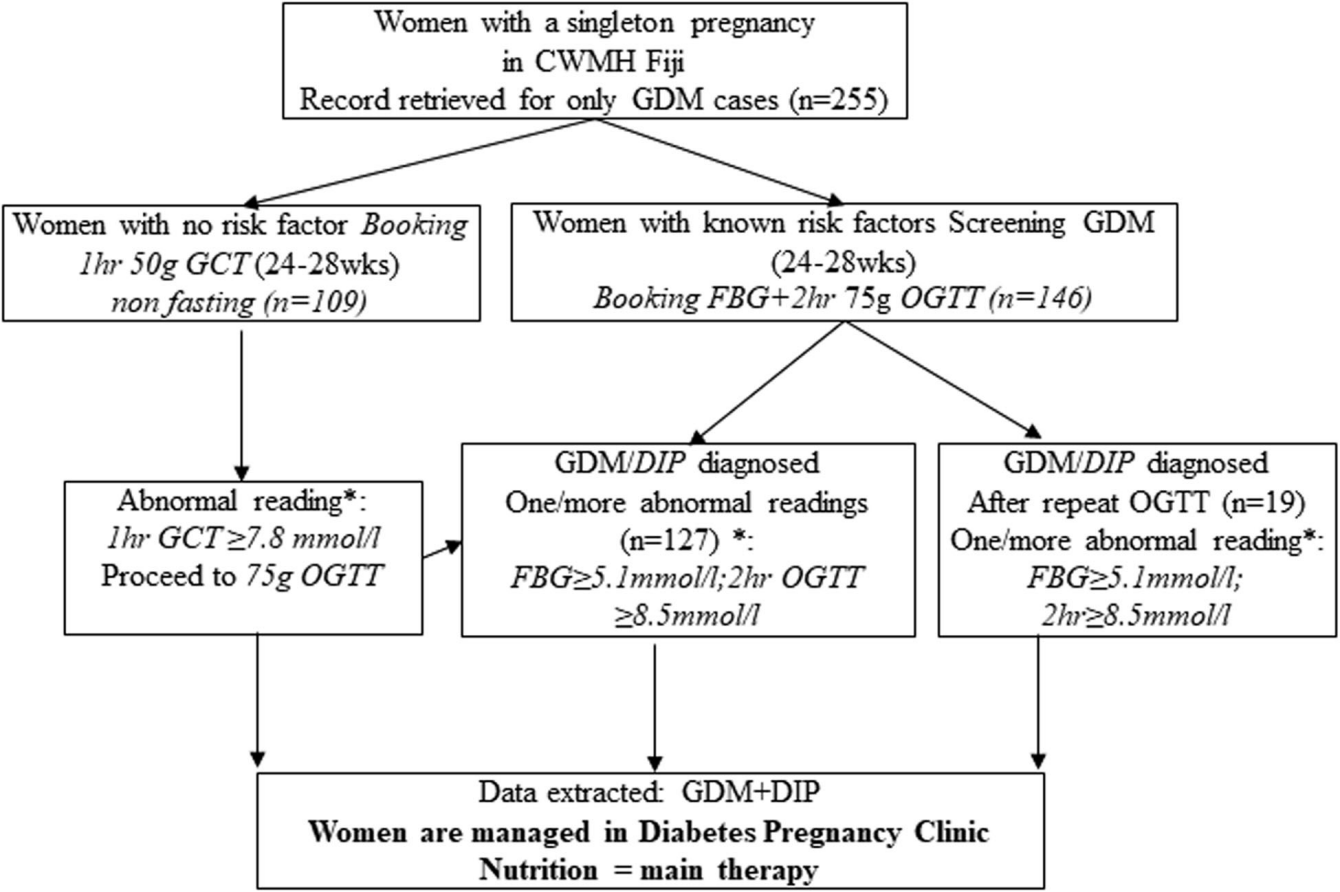
Flow chart for gestational diabetes mellitus (GDM) testing among pregnant women in an urban Fiji hospital. All pregnant women are routinely tested for GDM using a two-step process consisting of the 1-h glucose challenge test (GCT) at 24–28 weeks including a non-fasting 50 g glucose load and if GCT was ≥ 7.8 mmol/l, a 2-h 75 g oral glucose tolerance test (OGTT) was then performed. One abnormal value is sufficient for diagnosis. The women with any known risk factor for GDM including age ≥ 30 years, strong family history of diabetes, past history of GDM, previous macrosomic baby and high maternal pre-pregnancy BMI ≥ 30 kg/m^2^, proceed to OGTT at initial testing. Those who are at high risk for GDM (i.e. women with two or more of the risk factors present at booking) proceed directly to a 2-h 75 g OGTT at the time of their booking with the antenatal clinic. If the early testing with OGTT was normal (fasting < 5.1 mmol/L, 2 h < 8.5 mmol/L), the high-risk women underwent another 75-g OGTT at 24–28 weeks’ gestation

In the Colonial War Memorial Hospital (CWMH) Fiji, GDM was previously diagnosed using the ADIPS criteria which consisted of a 75 g glucose load with a fasting blood glucose ≥5.5 mmol/l and 2 h ≥8.0 mmol/l and only pregnant women with known risk factors for GDM such as previous GDM and previous big babies, were tested for GDM. In 2013, the hospital commenced a 2-step process of universal testing for GDM, using the modified International Association of Diabetes and Pregnancy Study Groups (IADPSG) criteria described in Fig. 1. Adoption of these criteria followed the findings of the landmark HAPO observational study in 2008 which reported significant relationship between maternal glycaemic levels and pregnancy outcomes [20]. Implementation of the modified (IADPSG) criteria was expected to not only increase the prevalence of GDM, but also to identify the population at higher risk of adverse pregnancy outcomes and who may benefit from targeted interventions to improve pregnancy outcomes [21].

Fijians are genetically different from other populations [22] and along with other Pacific Island nations make up eight of the world’s 10 most obese countries [23]. Pacific Islander women are heavier during pregnancy, have higher rates of macrosomia, higher incidence of abnormal glucose tolerance test results post-partum [17], and hence a higher risk of future development of type 2 diabetes mellitus compared to other ethnic groups [24].

This study was conducted to provide recent evidence on the prevalence of, and socio-demographic factors associated with, adverse neonatal outcomes among women with GDM in Fiji. The findings of this study are important to inform local policy and should enable public health researchers to target a sub-population of women with GDM for future interventions and allocation of local resources to areas with high need. The study is also likely to inform practice in rural/remote areas in Fiji and those with significant Melanesian and Polynesian populations (e.g. New Zealand and Australia).

## Subjects and methods

### Setting

Fiji is an island nation in the south-west Pacific Ocean, located between Vanuatu and Tonga. As at 2011, the country has a total population of approximately 884,887 (50.7% men, 49.3% women) which is comprised of about 57% Indigenous Fijians (iTaukei), 37% FIDs, and 6% others (including other Pacific people, Chinese and those of European descent). Indigenous Fijians are predominantly of Melanesian extraction, with some Polynesian admixture. About 56% of Fiji’s population resides in urban areas, with the Suva region being the most heavily populated [25]. Colonial War Memorial Hospital (CWMH) in Suva is Fiji’s largest and oldest hospital, and the national referral hospital for Fiji with services that are accessed by other Pacific Island countries [26].

### Data sources

Data for 255 women with GDM who gave birth to singleton infants at CWMH in Suva between June 2013 and May 2014, were retrieved from the Diabetes in pregnancy registry; the timeframe 2013–2014 was chosen because it was when the modified IADPSG [27] criteria for GDM diagnosis were introduced in the hospital. In our analysis, women with pre-existing diabetes i.e. those with known type 1 and type 2 diabetes, were excluded (Fig. 1).

### Sample size

The required sample size for this study was determined using a single population proportion formula. An earlier study reported in 1983 that the prevalence of GDM in Fiji was 22.7% [16]. This present study assumed the differences in the prevalence of GDM between urban and rural Fijians ranged between 5 and 7% [16], at 80% power and 5% significance level. This gives a sample of 251 women with GDM. Taking into account a dropout rate of about 2%, also based on earlier research [16], a total sample of approximately 255 participants was required. This sample size was sufficient to detect any statistical differences in examining factors associated with GDM among Fijian women.

### Outcome variables

**Fig. 2:**
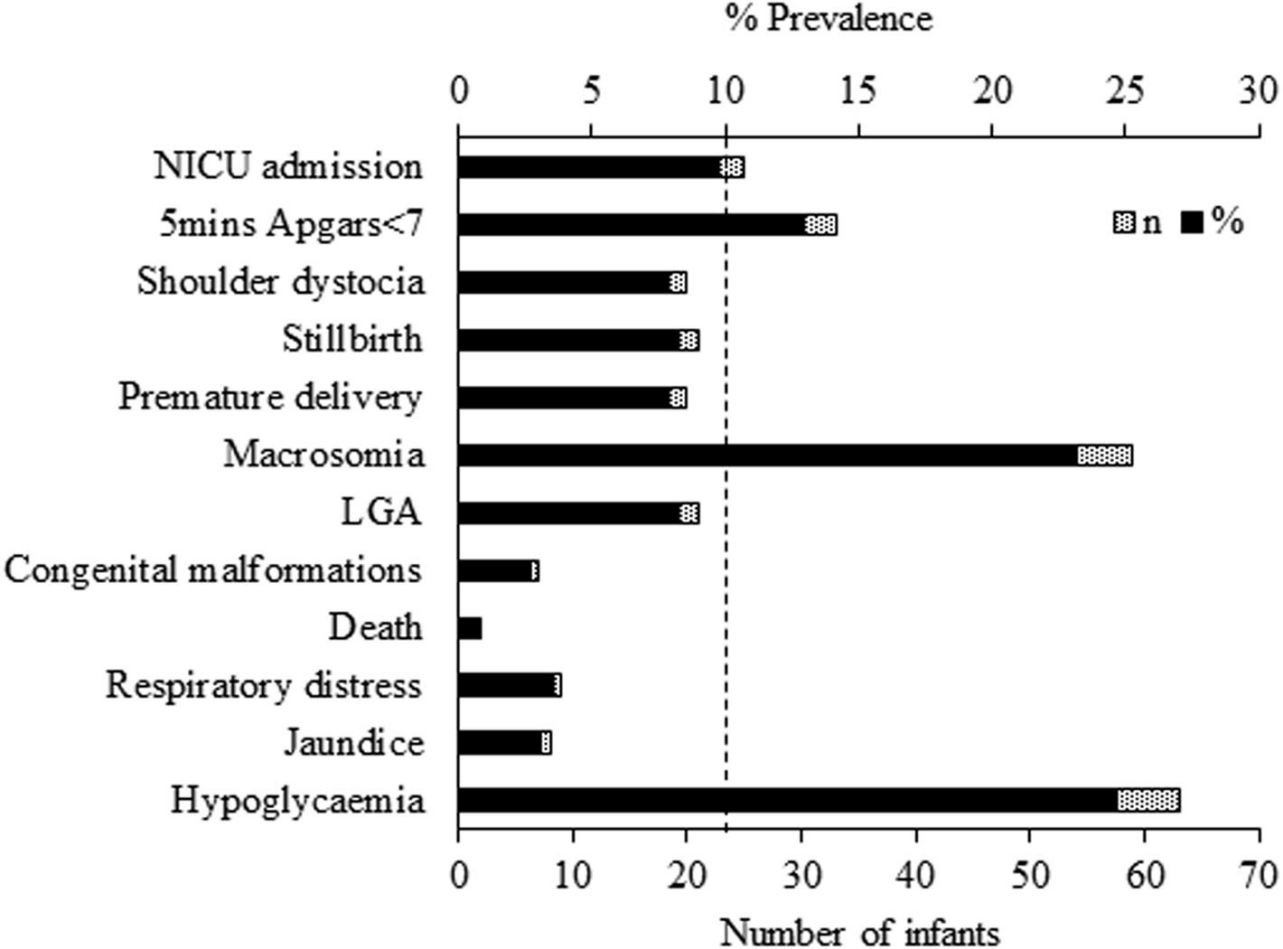
Prevalence of neonatal outcomes among Fijian women with gestational diabetes mellitus (GDM). LGA, large for gestational age

To determine the outcome variables, we conducted preliminary analysis using frequency distribution for all the neonatal outcomes (see dotted vertical line in Fig. 2) and only those with proportions > 10% were retained as final outcome variables. This was done to avoid the loss in precision of estimates with wide confidence intervals [28]. The three neonatal outcome variables that were retained in the analysis included: neonatal hypoglycaemia (which was defined as neonatal glucose ≤1.6 mmol/l during the first 24 h after birth [29]), macrosomia (baby weighing> 4 kg) [20] and Apgar score < 7 after 5 min. The outcome variables were coded as binary, ‘1’ for the presence and ‘0’ for absence. In our analysis, we combined data for those with GDM and ***DIP*** due to low count of women with *DIP*.

### Potential cofounding factors

The choice of potential cofounding factors was based on previous studies [30–32] and included socio-demographic factors (age, ethnicity, marital status, parity, level of education); maternal factors such as body mass index (BMI) calculated at the first prenatal visit using WHO criteria as: underweight (< 18.5 kg/m^2^), normal (18.5–24.9 kg/m^2^), and overweight (from 25 to 29.9 kg/m^2^), class 1 obesity (30–34.9 kg/m^2^) and class 2/3 obesity ≥35 kg/m^2^ [33], positive family history of diabetes, past history of GDM, baby weighing > 4 kg, stillbirth and neonatal death (which were simply recorded as present or absent); antenatal factors (gestational age at booking, gestational age at diagnosis and gestational age at delivery); and maternal complications such as preeclampsia (defined as hypertension that is onset from at least 20 weeks of pregnancy and accompanied by proteinuria), polyhydramnios, trauma, endometritis and wound infection and mode of delivery (caesarean section and vaginal delivery). Gestational age at delivery was classified into < 37 weeks and ≥ 37 weeks. In the regression analysis, BMI was further collapsed into three categories normal, overweight and obese due to low count of underweight, class 2 and class 3 obese women, and levels of education were classified into non-tertiary (no education, primary, secondary) and tertiary (university and polytechnic).

### Statistical analysis

Analyses involved frequency tabulations of all confounding factors in the study population. This was followed by cross tabulation to determine the prevalence of all potential confounding factors. Univariate logistic regression and multivariable logistic regression were performed to determine factors associated with the three key neonatal outcomes of macrosomia, neonatal hypoglycaemia and low Apgar score among offspring of women with GDM. The odds ratios with 95% confidence intervals were calculated to assess the adjusted odds of the independent variables. All analyses were carried out using STATA/MP version 14 (Stata Corp, College Station, TX, USA).

### Ethics

The study used existing datasets that are available from patients’ records, and all identifier information was removed prior to analysis. The study was approved by the Ethics Committee of the College of Medical Nursing and Health Sciences of the Fiji National University and by the Fiji National Health Research Ethics Review Committees (ref #: 2015.48.CEN).

## Results

### Characteristics of Fijian women with GDM

The majority were married (88.2%) women who were aged between 26 and 35 years (62.8%) and about one-third had tertiary education (37.2%). The sample had almost equal proportion of Itaukei Fijians and FIDs (49.4% vs 42.0%), the two main ethnic groups in Fiji. Table 1 shows the characteristics of women with GDM in CMWH Fiji. About one-half of mothers booked between 14 and 27 weeks of gestation. A hundred and seventy-three women (67.8%) had vaginal delivery (majority were non-assisted/normal vaginal deliveries (*n* = 165, 64.7%)) and 32.2% delivered by caesarean section. As indicated in Fig. 2, about 24.7% of the women with GDM had hypoglycaemic infants, 23.1% had macrosomic infants, and 12.9% had infants with Apgar scores < 7 at 5 min. Shoulder dystocia, stillbirth, premature delivery, large for gestational age (LGA) babies and NICU admissions were other serious adverse neonatal outcomes observed among Fijian women with GDM with prevalence < 10%, each.

**Table 1:** Maternal Characteristics of Fijian women with GDM

Study characteristics	*n* = 255	%
Maternal sociodemographic characteristics		
*Age (years)*	30.7 ± 5.5	
18–25	46	18.0
26–35	160	62.8
≥ 36	49	19.2
*Race/ethnicity*		
Others^§^	22	8.6
Itaukei Fijians	126	49.4
FIDs	107	42.0
*Educational level*^*ǂ*^		
Non-tertiary (primary/secondary)	157	62.8
Tertiary (university/polytechnic)	93	37.2
*Marital status*		
Not married	30	11.8
Married	225	88.2
*Gravidity (number of pregnancies)*		
Primigravid (first pregnancy)	34	13.3
Multiparous (2/more children)	177	69.4
Grand multiparous (≥5 children)	44	17.3
Maternal risk factors (yes, no)		
*Family history of diabetes*		
Yes	128	50.2
No	127	49.8
*Previous history of GDM*		
Yes	11	4.3
No	244	95.7
*Previous baby > 4 kg*		
Yes	55	21.6
No	200	78.4
*Previous stillbirth*		
Yes	11	4.3
No	244	95.7
*Previous neonatal death*		
Yes	10	3.9
No	245	96.1
*Maternal BMI (kg/m*^*2*^*)*^*ǂ*^	33.2 ± 7.5	
Underweight/normal (< 25)	35	14.9
Overweight (25–29.9)	40	17.0
Obese (classes 1–3, ≥30.0)	160	68.1
Antenatal factors *(weeks)*		
*Gestational age at booking*	18.9 ± 7.5	
1st trimester (0–13)	79	31.0
2nd trimester (14–27)	134	52.6
3rd trimester (≥28)	42	16.5
*Gestational age at GDM diagnosis*	25.6 ± 7.8	
1st trimester (0–13)	26	10.2
2nd trimester (14–27)	111	43.5
3rd trimester (≥27)	118	46.3
*Gestational age at delivery*	38.3 ± 2.2	
< 37	44	17.2
37–41	211	82.8
Maternal post-pregnancy factors		
*Mode of delivery*		
Vaginal delivery	173	67.8
Caesarean section	82	32.2
*Induced labour*		
Yes	98	38.4
No	157	61.6
*Preeclampsia*		
Yes	48	18.8
No	207	81.2
*Polyhydramnios*		
Yes	2	0.8
No	253	99.2
*Infection*^*ǂ*^		
Yes	3	1.2
No	251	98.8

*BMI* body mass index, *GDM* gestational diabetes mellitus, *FIDs* Fijians of Indian Descent

^§^others means part-Europeans, Chinese, and other Pacific Islanders

^ǂ^there were few missing data, so the denominator was less than 255

### Factors associated with macrosomia among Fijian women with GDM

Table 2 shows the prevalence, univariate and multivariable regression analysis of factors associated with neonatal macrosomia. As indicated in the table, univariate analysis indicated that race or ethnicity, marital status, previous baby weighing > 4 kg, gestational age at booking, gestational age at GDM diagnosis and gestational age at delivery were significantly associated with neonatal macrosomia. After adjusting for potential confounding factors, our results revealed that marital status (married) and having a previous baby weighing > 4 kg were significantly associated with neonatal macrosomia.

**Table 2:** Prevalence, unadjusted (OR) and odds ratios (AOR) for neonatal macrosomia among Fijian women with GDM, 2013–2014

Variables	Prevalence of macrosomia (%)	OR	95% CI	AOR	95% CI
Maternal sociodemographic characteristic					
*Age (years)*					
18–25	17.4	1.00		1.00	
26–35	25.0	1.58	0.68, 3.68	1.27	0.44, 3.72
≥ 36+	22.5	1.38	0.50, 3.80	0.70	0.17, 2.89
*Race/ethnicity*					
Others^§^	31.8	1.00		1.00	
Itaukei Fijians	31.0	0.96		0.95	0.29, 3.12
FIDs	12.2	0.30	0.10,0.80^*^	0.50	0.12, 2.02
*Educational level*					
Non-tertiary (primary/secondary)	24.2	1.00		1.00	
Tertiary (university/polytechnic)	21.5	0.86	0.46, 1.59	0.64	0.29, 1.42
*Marital status*					
Not married	40.0	1.00		1.00	
Married	20.9	0.40	0.16, 0.88^*^	0.29	0.11, 0.77^*^
*Gravidity (number of pregnancies)*					
Primigravid (first pregnancy)	11.8	1.00		1.00	
Multiparous (2/more children)	23.7	2.33	0.78, 7.00	1.17	0.30, 4.63
Grand multiparous (≥5 children)	29.6	3.15	0.92, 10.74	0.85	0.15, 4.69
Maternal risk factors (Yes/No)					
*Family history of diabetes*					
No	28.4	1.00		1.00	
Yes	17.8	0.55	0.31, 1.00	0.69	0.31, 1.54
*Previous history of GDM*					
No	23.4	1.00		1.00	
Yes	18.2	0.73	0.15, 3.47	3.68	0.58, 23.40
*Previous baby > 4 kg*					
No	15.5	1.00		1.00	
Yes	50.9	5.65	2.94, 10.86^*^	6.08	2.46, 15.01^*^
*Previous stillbirth*					
No	23.4	1.00		1.00	
Yes	18.2	0.73	0.15, 3.47	0.51	0.07, 3.70
*Previous neonatal death*					
No	22.9	1.00		1.00	
Yes	30.0	1.45	0.36, 5.78	2.92	0.46, 18.72
*Maternal BMI (kg/m*^*2*^*)*					
Underweight / normal)	17.1	1.00		1.00	
Overweight	15.0	0.85	0.25, 2.93	0.93	0.21, 4.12
Obese (classes 1–3, ≥30.0)	26.3	1.72	0.67, 4.43	1.11	0.33, 3.71
Antenatal factors					
*Booking age of gestation (weeks)*					
1st trimester (0–13)	12.7	1.00		1.00	
2nd trimester (14–27)	26.9	2.53	1.18, 5.45^*^	1.59	0.60, 4.25
3rd trimester (≥28)	31.0	3.09	1.22, 7.85^*^	0.67	0.18, 2.57
*Gestational age at GDM diagnosis (weeks)*					
1st trimester (0–13)	3.9	1.00		1.00	
2nd trimester (14–27)	21.6	6.90	0.89, 53.53	3.44	0.34, 34.81
3rd trimester (≥27)	28.8	10.12	1.32, 77.67^*^	4.30	0.41, 45.30
Gestational age at delivery (weeks)					
< 37	9.1	1.00		1.00	
37–41	26.1	3.53	1.21, 10.31^*^	2.72	0.75, 9.86
Maternal post-pregnancy factors					
*Delivery intervention*					
*Induced labour*					
No	23.3	1.00		1.00	
Yes	23.1	0.99	0.18, 0.50	1.37	0.13, 14.30
*Caesarean section*					
No	24.3	1.00		1.00	
Yes	20.5	0.80	0.42, 1.54	1.16	0.55, 3.49
*Preeclampsia*					
No	23.2	1.00		1.00	
Yes	22.9	0.98	0.47, 2.08	1.13	0.43, 3.02

^*^Confidence Interval (CI) that does not include 1.00, significant

*BMI* body mass index, *GDM* gestational diabetes mellitus, *FIDs* Fijians of Indian Descent

^§^others means part-Europeans, Chinese, and other Pacific Islanders

### Factors associated with neonatal hypoglycaemia among Fijian women with GDM

The prevalence, univariate and multivariable regression analysis of factors associated with neonatal hypoglycaemia are presented in Table 3. From the table, it can be seen from the univariate analysis that maternal BMI and gestational age at delivery were significantly associated with neonatal hypoglycaemia. After adjusting for potential confounding factors, our results revealed that gestational age at delivery (< 37 weeks of gestation) and maternal BMI (overweight, BMI of 25–29.9 kg/m^2^) were significantly associated with neonatal hypoglycaemia.

**Table 3:** Prevalence, unadjusted (OR) and odds ratios (AOR) for neonatal hypoglycaemia among Fijian women with GDM, 2013–2014

Variables	Prevalence of hypoglycaemia (%)	OR	95% CI	AOR	95% CI
Maternal sociodemographic factors					
*Age (years)*					
18–25	21.7	1.00		1.00	
26–35	22.6	1.05	0.48, 2.33	0.82	0.32, 2.14
≥ 36	34.7	1.91	0.77, 4.77	1.31	0.40, 4.35
*Race/ethnicity*					
Others^§^	31.8	1.00		1.00	
Itaukei Fijians	26.2	0.76	0.29, 2.03	0.97	0.30, 3.13
FIDs	21.7	0.59	0.22, 1.63	0.95	0.26, 3.47
*Educational level*					
Non-tertiary (primary/secondary)	26.9	1.00		1.00	
Tertiary (university/polytechnic)	21.5	0.74	0.41, 1.37	0.76	0.37, 1.56
*Marital status*					
Not married	27.6	1.00		1.00	
Married	24.4	0.85	0.36, 2.03	0.72	0.26, 1.97
*Gravidity (number of pregnancies)*					
Primigravid (first pregnancy)	11.8	1.00		1.00	
Multiparous (2/more children)	27.3	2.81	0.94, 8.40	2.42	0.67, 8.73
Grand multiparous (≥5 children)	25.0	2.50	0.72, 8.70	1.46	0.29, 7.42
Maternal risk factors (yes, no)					
*Family history of diabetes*					
No	26.8	1.00		1.00	
Yes	22.8	0.81	0.46, 1.43	0.95	0.47, 1.95
*Previous history of GDM*					
No	24.3	1.00		1.00	
Yes	36.4	1.78	0.50, 6.30	0.83	0.16, 4.39
*Previous baby > 4 kg*					
No	22.6	1.00		1.00	
Yes	32.7	1.66	0.87, 3.20	1.19	0.51, 2.76
*Previous stillbirth*					
No	24.7	1.00		1.00	
Yes	27.3	1.14	0.29, 4.45	1.42	0.27, 7.45
*Previous neonatal death*					
No	24.2	1.00		1.00	
Yes	40.0	2.09	0.57, 7.66	3.96	0.79, 19.90
*Maternal BMI (kg/m*^*2*^*)*					
Underweight/normal (< 25)	8.8	1.00		1.00	
Overweight (25–29.9)	32.5	4.98	1.28, 19.33*	5.49	1.01, 29.96*
Obese (classes 1–3, ≥30.0)	27.5	3.92	1.14, 13.48*	4.34	0.87, 21.65
Antenatal factors					
*Booking age of gestation (weeks)*					
1st trimester (0–13)	24.1	1.00		1.00	
2nd trimester (14–27)	25.6	1.08	0.57, 2.07	1.72	0.68, 4.34
3rd trimester (≥28)	23.8	0.99	0.41, 2.37	1.09	0.30, 3.95
*Age of gestation at GDM diagnosis (weeks)*					
1st trimester (0–13)	38.5	1.00		1.00	
2nd trimester (14–27)	21.6	0.44	0.18, 1.10	0.40	0.10, 1.52
3rd trimester (≥27)	24.8	0.53	0.22, 1.29	0.53	0.13, 2.17
*Age of gestation at delivery (weeks)*					
< 37	39.5	1.00		1.00	
37–41	21.8	0.43	0.21, 0.85*	0.38	0.17, 0.89*
Maternal post-pregnancy factors					
*Delivery intervention*					
*Induced labour*					
No	23.3	1.00		1.00	
Yes	25.6	1.14	0.62, 2.09	0.52	0.06, 4.20
*Caesarean section*					
No	21.0	1.00		1.00	
Yes	33.3	1.88	1.04, 3.40	2.86	0.15, 56.32
*Preeclampsia*					
No	22.8	1.00		1.00	
Yes	33.3	1.69	0.86, 3.35	1.32	0.59, 3.01
*Polyhydramnios*					
No	24.6	1.00		1.00	
Yes	50.0	3.06	0.18, 49.72	2.32	0.11, 48.31
*Endometritis*					
No	24.7	1.00		–	
Yes	50.0	3.05	0.19, 49.5	–	–

^*^Confidence Interval (CI) that does not include 1.00, significant

*BMI* body mass index, *GDM* gestational diabetes mellitus, *FIDs* Fijians of Indian Descent

^§^others means part-Europeans, Chinese, and other Pacific Islanders

### Prevalence and factors associated with neonatal low Apgar Score < 7 at 5 min

Table 4 shows the prevalence, univariate and multivariable regression analysis of factors associated with neonatal Apgar score < 7 at 5 min. As indicated in the table, univariate analysis indicated that gestational age at delivery was significantly associated with neonatal low Apgar score. After adjusting for potential confounding factors, our results revealed that gestational age at booking (≥28 weeks) and at delivery (< 37 weeks), and maternal pre-eclampsia, were significantly associated with neonatal low Apgar score among Fijian women with GDM.

**Table 4:** Prevalence, unadjusted (OR) and odds ratios (AOR) for lower Apgar scores among Fijian women with GDM, 2013–2014

Variables	Prevalence of low Apgar score (%)	OR	95% CI	AOR	95% CI
Maternal sociodemographic factors					
*Age (years)*					
18–25	15.2	1.00		1.00	
26–35	13.9	0.90	0.36, 2.27	0.97	0.26, 3.59
≥ 36	8.2	0.50	0.14, 1.82	0.18	0.02, 1.44
*Race/ethnicity*					
Others^a^	9.1	1.00		1.00	
Itaukei Fijians	11.2	1.26	0.27, 5.98	1.32	0.20, 8.61
FIDs	16.0	1.91	0.41, 8.94	1.60	0.21, 11.92
*Educational level*					
Non-tertiary (primary/secondary)	16.0	1.00		1.00	
Tertiary (university/polytechnic)	8.7	0.50	0.22, 1.16	0.54	0.18, 1.62
*Marital status*					
Not married	10.3	1.00		1.00	
Married	13.4	1.34	0.38, 4.70	1.66	0.35, 7.85
*Gravidity (number of pregnancies)*					
Primigravid (first pregnancy)	11.8	1.00		1.00	
Multiparous (2/more children)	14.3	1.25	0.41, 3.85	2.77	0.58, 13.29
Grand multiparous (≥5 children)	9.1	0.75	0.17, 3.24	1.77	0.19, 16.60
Maternal risk factors (yes, no)					
*Family history of diabetes*					
No	13.4	1.00		1.00	
Yes	12.7	0.94	0.45, 1.96	1.40	0.52, 3.76
*Previous baby > 4 kg*					
No	13.6	1.00		1.00	
Yes	10.9	0.78	0.30, 1.99	1.64	0.45, 5.95
*Previous neonatal death*					
No	13.2	1.00		1.00	
Yes	10.0	0.73	0.09, 5.98	1.49	0.12, 18.50
*Maternal BMI (kg/m*^*2*^*)*					
Underweight/normal (< 25)	14.7	1.00		1.00	
Overweight (25–29.9)	22.5	1.68	0.50, 5.62	1.58	0.37, 6.70
Obese (classes 1–3, ≥30.0)	10.7	0.69	0.24, 2.03	0.28	0.07, 1.16
Antenatal factors					
*Booking age of gestation (weeks)*					
1st trimester (0–13)	11.4	1.00		1.00	
2nd trimester (14–27)	12.9	1.15	0.49, 2.72	1.46	0.44, 4.85
3rd trimester (≥28)	16.7	1.56	0.54, 4.53	7.87	1.11, 55.75*
*Age of gestation at GDM diagnosis (weeks)*					
1st trimester (0–13)	11.5	1.00		1.00	
2nd trimester (14–27)	15.5	1.40	0.38, 5.19	1.53	0.24, 10.01
3rd trimester (≥27)	11.1	0.96	0.25, 3.64	0.53	0.06, 4.56
*Age of gestation at delivery (weeks)*					
< 37	27.9	1.00		1.00	
37–41	10.0	0.29	0.13, 0.64*	0.14	0.04, 0.46*
Maternal post-pregnancy factors					
*Delivery intervention*					
*Induced labour*					
No	10.6	1.00		1.00	
Yes	14.3	1.41	0.62, 3.18	0.16	0.01, 2.52
*Caesarean section*					
No	15.4	1.00		1.00	
Yes	7.7	0.46	0.18, 1.16	2.86	0.16, 56.32
*Preeclampsia*					
No	11.2	1.00		1.00	
Yes	20.8	2.08	0.92, 4.73	5.57	1.73, 18.00*
*Polyhydramnios*					
No	12.8	1.00		1.00	
Yes	50.0	6.84	0.42, 112.15	10.51	0.34, 326.38

^*^Confidence Interval (CI) that does not include 1.00, significant

*BMI* body mass index, *GDM* gestational diabetes mellitus, *FIDs* Fijians of Indian Descent

^§^others means part-Europeans, Chinese, and other Pacific Islanders

## Discussion

In the last 30 years, this is the first study to provide evidence of GDM and associated outcomes among Fijian women. We found high rates of adverse neonatal outcomes in this population which far exceeded the rates for the background population and in some cases almost doubled the rate [34] and they were far above the rates among other Pacific Islander women [32, 35, 36]. The key factors associated with such high rates of neonatal outcomes among Fijian women with GDM included previous baby weighing > 4 kg, maternal BMI (overweight/obese), gestational age at booking (≥28 weeks) and gestational age at delivery (< 37 weeks), and maternal pre-eclampsia or pregnancy-induced hypertension. Also of importance in this population were obesity, ethnicity, and a family history of diabetes, which interacted with other variables to influence the odds of neonatal outcomes in this study.

The proportion of women who had macrosomic babies (23%) in this study (see Fig. 2) was comparable to the global report in GDM (15–45%) but the rates almost doubled the reported rate among other Pacific Islands women with [32, 35–38] and without GDM (12%) [39]. This may be related to the high rate of obesity in this cohort (where 68% of the women were obese, Table 1) - a reflection of the obesity epidemic in the country. The finding of a significant association between macrosomia and previous baby weighing > 4 kg was consistent with previous reports from observational [40, 41] and cohort studies [42] that showed a strong relationship between macrosomia and a previous history of macrosomia. In the observational study, the authors found that up to 78% of the women who reported a previous history of macrosomia had repeat macrosomia [41]. It is known that all macrosomic infants represent a high-risk group, regardless of maternal diabetes status [43, 44]. Clausen et al. in 2005 [45] and Schaefer-Graf in 2003 [46] reported similar data. They found that high HbA_1c_ at admission and maternal BMI were independently associated with serious adverse fetal outcomes including perinatal mortality and/or major congenital malformations [45], and maternal BMI and previous macrosomic baby appear to have the strongest influence on fetal growth in the late second and early third trimester; maternal glycaemia predominated in the third trimester [46]. It was suggested that the process leading to macrosomia in infants starts in the second trimester of pregnancy [47], however, other studies have found a relationship of some factors in 1st trimester that correlated with macrosomia [48, 49]. These findings suggest the need for early identification of the women with GDM (particularly those at high risk) for interventions that could potentially reduce the rate of macrosomia and other outcomes associated with macrosomia such as shoulder dystocia and caesarean section deliveries which were also higher in this study compared to the background population [50].

Although the prevalence of neonatal hypoglycaemia was higher in this study than in previous studies involving European (3–4%) [35, 51] and other Pacific Islander (16.6%) women with GDM [32, 35], the association with maternal BMI was consistent with reports from Brazil [42], Spain [42], Australia [43] and New Zealand [29, 34] among women with GDM. The burden of adult overweight, obesity, and associated non-communicable diseases (NCDs) has been widely acknowledged in at least 17 Pacific Island countries, including Fiji, which have current national NCD plans or strategies in place [52]. The findings of this study suggest the need for a pre-pregnancy program that incorporates both lifestyle and dietary interventions in addressing the issue of obesity among Fijian women with GDM [53].

Another important finding of this study was the significant reduction in the odds of all three key outcomes particularly for neonatal hypoglycaemia and low Apgar score, among women who delivered at term (38–41 weeks of gestation) compared to those who delivered before 37 weeks of gestation. Similar association between pre-term delivery and hypoglycaemia have been reported not only among women with GDM [54, 55] but also among those without GDM [56] and the risk increased with the level of hyperglycaemia [57]. The rate of preterm delivery in this cohort (17%) was almost double the rate that was reported for the background population (9.9%) in 2012 [34]. In this study, infants who were delivered at term were less likely to have low Apgar score compared to infants who were delivered preterm. This finding may be related to the fact that fewer women had preterm or caesarean deliveries compared with those who had delivered at term or by vaginal delivery (Table 1). A previous study found that low Apgar scores at 5 min was more frequent in infants who were delivered preterm or by caesarean section compared with infants who were delivered at term or by vaginal delivery [58].

There were other serious adverse outcomes in this study such as shoulder dystocia, stillbirths, and NICU admissions (Fig. 2) which were also worse in this population compared to other Pacific Islands women with GDM [11, 32, 35, 36]. These may reflect the higher burden of the disease [59] as well as the low quality of obstetric care in public hospitals in Fiji. Ensuring that pregnant women with GDM, particularly those at high risk of complications, are capable of self-monitoring their glucose levels by making available home blood glucose meters (which currently is not possible due to lack of affordability) will help improve pregnancy outcomes in GDM. Additionally, the high rate of low Apgar scores found in this study indicates the need for regular audit of the diabetes clinic to identify areas of improvement in the quality of care.

Pacific Islands women are known to book late in gestation [9] and this was reflected in our study where more than two-thirds of the women had late booking (booked after 13 weeks of gestation). In a recent randomised RCT, early booking in gestation identified pregnant women who can benefit from early treatment for GDM, to reduce pregnancy complications [60]. In the TOBOGM study, 89% of untreated women had GDM early in gestation (at both 18 weeks and 24–28 weeks gestation), making the case for early screening in pregnancy [60], however, the decision as to whether to treat or not early in gestation remains controversial. The present study found that early booking in gestation was associated with some reduction in the odds of macrosomia and significant reduction in the odds of low Apgar score by up to 80% (Table 4). Public health officers need to step up effective targeted strategies to promote early booking in gestation preferably in the first trimester of pregnancy, among women with and without GDM.

The limitations of this study were that: 1), the findings cannot be generalized to the entire population of pregnant women with GDM in Fiji because we only considered women with GDM from a single hospital registry in urban Fiji; 2), the study reported outcomes of mothers with GDM without a control group for direct comparison. Future studies should compare the sociodemographic factors associated with adverse outcomes between women with and without GDM; 3), the study did not adequately test the effect of pre-pregnancy BMI and data for gestational weight gain was not captured. However, with the high prevalence of overweight and obesity in this study, it is critical to understand the effect of maternal body mass on neonatal outcomes; 4), although the use of glucose challenge tests for screening those with no risk factors is an acceptable approach [61], the test potentially misses those with fasting hyperglycaemia, thus OGTT was performed in this study; and 5), the study used a cut-off point of < 1.6 for neonatal hypoglycaemia that should be considered when interpreting the results. Despite these limitations, the high prevalence of key neonatal outcomes of macrosomia and hypoglycaemia, may not have been different in this study, since previous studies have reported similar worse outcomes among women with GDM who were under strict glycaemic control [32, 35]. Similar to some studies [11, 62], we used the *modified* IADPSG criteria since the 1 h glucose was not available, which may have underestimated the odd ratios as more women with GDM could have been diagnosed using a 1 h blood glucose test compared to the 2-h glucose test (36% versus 13%) [63]. To be able to assess the impact of the IADPSG criteria in our setting, future studies should compare the maternal and fetal outcomes of women with GDM who were diagnosed using a 1 h blood glucose test and those diagnosed using the 2-h glucose test, to provide an opportunity for assessment of the cost effectiveness of both approaches.

This study has some strengths including inclusion of other clinical variables in the multivariable analysis, providing baseline information with which the ongoing universal testing can be assessed and allow endorsement of awareness campaigns to provide more knowledge on the adverse effects of GDM on neonates. The maternal socio-demographic characteristics as well as factors identified in this study can be used to develop future interventions to optimize maternal and infant health outcomes among Fijian women with GDM. The findings alert the health care providers on the high rate of macrosomia, hypoglycaemia and low Apgar Score in an urban Fiji population, and can alert Fijian women of their increased risk of adverse neonatal outcomes. While there are no published hyperglycaemia in pregnancy data from Fiji, this study showed worse birth outcomes compared to our earlier work (1994–1998) among Polynesians (including Fijians) and Europeans in South Auckland, New Zealand [35] which may be attributed to the anthropomorphic effects of the Fijian population. Given that 80% of births in the Central Eastern division occur in CWM hospital, it is likely that these findings may represent the Central Eastern division population.

## Conclusions

This study has shown that offsprings of Fijian women with GDM have serious negative outcomes specifically macrosomia, hypoglycaemia and Apgar score. The risk increases among overweight/obese women, women with a previous baby weighing > 4 kg, had delivered pre-term babies, had pre-eclampsia and those who booked later than 13 weeks in gestation. These factors should be taken into account when preventive intervention strategies are developed to improve outcomes and the target risk group is established. The high incidence of complications reported in this paper is clear evidence of the burden of GDM. Public enlightenment campaigns promoting booking in the first trimester among women with GDM and effective lifestyle interventions to prevent excessive weight gain in pregnancy are needed to improve the outcomes of pregnancies with GDM in the short term, and to reduce the long-term risk of type 2 diabetes for both mothers and their children.

## Data Availability

The datasets generated and analyzed during the current study are not publicly available due to the hospital policy but are available from the corresponding author on reasonable request.

## Abbreviations

AOR: Adjusted odds ratio
CWMH: Colonial War Memorial Hospital
*DIP*: Diabetes in pregnancy
GDM: Gestational diabetes mellitus
IADPSG: International Association of Diabetes and Pregnancy Study Groups
WHO: World Health Organization
